# How health beliefs and sense of control predict adherence to COVID-19 prevention guidelines among young adults in South Korea

**DOI:** 10.3389/fpsyg.2022.1025638

**Published:** 2022-12-15

**Authors:** Gayeon Lee, Soo Hyun Park

**Affiliations:** Department of Psychology, Yonsei University, Seoul, South Korea

**Keywords:** COVID-19, sense of control, health belief model (HBM), perceived susceptibility, perceived severity

## Abstract

This study defined adherence to COVID-19 prevention guidelines as health behavior and examined whether the two constructs of the health belief model (i.e., perceived susceptibility and perceived severity) and sense of control predict the level of adherence among young adults in South Korea. An online survey (*N* = 200) conducted in June 2021, showed that perceived susceptibility, perceived severity, and sense of control positively predict adherence behavior. Sense of control significantly moderated the relationship between perceived susceptibility and adherence even after controlling for depression and perceived health status. Specifically, individuals with a lower level of perceived susceptibility still adhered to COVID-19 prevention guidelines if they had a higher level of sense of control. The finding demonstrates the key role of sense of control in promoting adherence to COVID-19 prevention guidelines and the relationship between sense of control and two constructs of the health belief model. Implication for public messaging targeted at young adults during pandemic situations is also discussed.

## Introduction

The unprecedented COVID-19 pandemic situation has restricted almost every aspect of people’s lives. While vaccination is considered one of the strongest ways to prevent COVID-19 infection (Mancuso et al., 2021), and it has become widely available, many governments have also emphasized the importance of adhering to other preventive measures, such as social distancing, defined as keeping a physical distance from other people in order to prevent the transmission of the virus (Callaway, 2020; Krause et al., 2021; Shahcheraghi et al., 2021). The US Centers for Disease Control and Prevention (CDC) recommends that people stay six feet away from each other, wear a mask, and wash their hands frequently. Its South Korean counterpart, the Korea Disease Control and Prevention Agency (KDCA), encourages people to stay at least two meters (6.56 feet) away from each other, cover their mouths when coughing, and to avoid touching their faces with unwashed hands.

Although social distancing has played a part in decreasing the COVID-19 infection rate (Thu et al., 2020; Wellenius et al., 2021), there are still many people who do not comply with public health recommendations (Pedersen and Favero, 2020; Hills and Eraso, 2021; Williams et al., 2021). In South Korea, religious gatherings that occurred in the city of Daegu in violation of the country’s COVID-19 prevention guidelines, including those regarding social distancing, resulted in a noticeable spike in the number of confirmed cases. Other spikes also occurred when people attended crowded clubs in Itaewon, a popular social gathering location in South Korea. At least 277 people were infected as a result of one Itaewon club cluster infection which involved people singing and shouting without masks in violation of social distancing rules (Korea Disease Control and Prevention Agency, 2020). These examples beg the question of why some individuals do not adhere to COVID-19 prevention guidelines, including the social distancing rules. Thus, the present study examined how certain factors influenced health behaviors, with adherence to KDCA guidelines as the key variable of interest.

A study revealed that participants aged 15–30 had reported twice as much contact with other people compared to participants who were 50 years old and older (Canning et al., 2020). In addition, Pedersen and Favero (2020) found that participants who were more than 45 years old better adhered to social distancing policies than those who were younger. These results may have been due to the fact that younger people place more value on relationships than individuals in other developmental stages of life, which might also influence their decision-making (Altikulaç et al., 2019). Another factor to consider is that since younger individuals are relatively less susceptible to severe complications that can arise from contracting COVID-19, they may experience a social dilemma about adhering to preventive guidelines for the sake of others (Franzen and Wöhner, 2021). Therefore, this study targeted young adults because adherence may differ among different age groups with younger individuals experiencing more difficulty in adhering to preventive guidelines. Also, we specifically targeted young adults between the ages of 19–29 because the period overlaps with emerging adulthood, which is known to represent a discrete developmental period (Auerbach et al., 2018).

During the current COVID-19 pandemic situation, adherence to the COVID-19 prevention guidelines can be labeled a health behavior (Bourassa et al., 2020). Health behavior refers to the actions which individuals take to maintain or improve their health (Cockerham, 2014). Thus, washing hands, keeping a distance from other people, and wearing masks can be conceptualized as health behaviors related to COVID-19 which protect individuals from the possibility of infection. While many theories attempt to explain why people are willing or unwilling to engage in health behaviors, one of the most widely used theoretical models is the health belief model (HBM; Rosenstock, 1960, 1974). There are many constructs in the model including perceived susceptibility, perceived severity, perceived benefits, perceived barriers, and cue to action (Glanz et al., 2008). Among these constructs, perceived susceptibility, which is the degree to which someone thinks they will experience a given disease, strongly affects whether that individual will take measures to protect themselves against contracting that disease. Much extant research has focused on the effects of perceived susceptibility on promoting health behaviors during pandemics (Bish and Michie, 2010; Liao et al., 2010; Breakwell et al., 2021a; Venema and Pfattheicher, 2021). According to one study, high perceived susceptibility to contracting COVID-19 was associated with higher levels of vaccination intention (Wong et al., 2020). In another study, the scores on the COVID-19 Own Risk Appraisal Scale (CORAS) scores were positively correlated with the COVID-19 preventive behaviors indices (Breakwell et al., 2021b). Furthermore, perceived severity, which is how severe someone thinks a disease’s symptoms and aftereffects are, affects people’s health behaviors during pandemics as well (Bish and Michie, 2010; Lin et al., 2014). In one study, the difference in the level of preventive measures taken against COVID-19 between the younger and older generations decreased as the perceived severity of infection increased (Luo et al., 2021). Thus, as past studies on adherence to COVID-19 and other pandemics prevention guidelines attempted to shed light on how traditional HBM constructs affect adherence behaviors, we examined whether the HBM constructs also predicted young adults’ adherence to COVID-19 prevention guidelines along with other variables of interest.

Although HBM has been applied extensively in determining the underlying mechanism of various health behaviors engagement, some studies argued that each construct in HBM exhibits a small effect size on health behaviors (Orji et al., 2012). One meta-analysis result showed that perceived severity and susceptibility are relatively weaker predictors of health behaviors engagement than other HBM constructs and they may only have an indirect effect on health behaviors as described in the extended parallel model (Carpenter, 2010). The extended parallel model (EPPM; Witte, 1992), which explains how people control fear and danger, suggests that self-efficacy related to a particular behavior of fear control can moderate the effect of perceived severity and susceptibility on fear control behavior. Self-efficacy is usually defined as one’s capacity to perform *certain behaviors* to attain the desired result (Bandura, 1977, 1986, 1997). However, since the current pandemic situation is unique in that it has put severe constraints on almost every aspect of human activity, examining *control belief over life in general* might provide a better explanation on behavioral adjustment during the COVID-19 pandemic. Past studies showed that control belief over life, usually referred to as sense of control, can be lowered during a disaster situation (e.g., Afifi et al., 2014), and it affects one’s level of stress and depression and in turn, behavioral adjustment during a disaster (Sneath et al., 2009). Thus, this study examined whether sense of control moderated the effect of perceived susceptibility and perceived severity on adherence to COVID-19 prevention guidelines.

Sense of control is the degree to which individuals feel that they can control factors that affect many aspects of their lives (Pearlin et al., 1981). Although many factors such as age and education are known to influence sense of control (Mirowsky, 1995; Slagsvold and SØrensen, 2008), a high sense of control seems to predict better health, even after controlling for the influence of personality and social support (Ward, 2013). A recent 4-year longitudinal study reported that individuals who were in the highest quartile of sense of control not only reported better physical health outcomes–i.e., lower risk of strokes, cognitive impairments, chronic pains, and others–but also an overall better engagement in health-promoting behaviors such as increased physical activity and fewer sleep problems than those in the lower quartiles (Hong et al., 2021). In contrast, a lower level of sense of control is known to be associated with health-harming behaviors such as less self-initiated preventive care (Seeman and Seeman, 1983), addictive social media use (Brailovskaia and Margraf, 2021), and more alcohol-related problems (Surgenor et al., 2006).

During the COVID-19 pandemic, sense of control was negatively correlated with repetitive negative thinking (Brailovskaia and Margraf, 2021) as well as conspiracy theory endorsement (Šrol et al., 2021), and moderated the negative effects of regional pandemic severity on the psychological distance to COVID-19 (Zheng et al., 2020). In addition, Zhu et al. (2020) suggest that sense of control can be a crucial factor in determining the degree of adjustment to the pandemic situation and may also mediate the relationship between uncertain threats and behavioral adjustment. While they did not empirically test their assumption, past research shows that individuals experience a high level of anxiety and depression if there is a gap between the degree of perceived control and desired level of control (Moulding and Kyrios, 2006). With the current pandemic putting severe constraints on people’s behaviors, it is possible their sense of control may be affected, thereby influencing their behavioral adjustment to the COVID-19 situation. Thus, this study examined how sense of control was associated with adherence to prevention guidelines.

Along with HBM constructs and sense control, this study also examined how depression and anxiety affect adherence to COVID-19 prevention guidelines. An individual’s level of depression is negatively associated with health-promoting behaviors (Leas and McCabe, 2007; Dirmaier et al., 2010; Tan and Yadav, 2013; Susin et al., 2016) but positively with health risking behaviors (Teychenne et al., 2010; Asarnow et al., 2014). Anxiety also predicts a lower level of engagement in some health behaviors (Strine et al., 2005; Hohls et al., 2020). Moreover, depression and anxiety levels are negatively correlated with sense of control (Steptoe et al., 2007; Gallagher et al., 2014). Hence, this study examined how levels of depression and anxiety influence the relationship between sense of control and adherence to prevention guidelines, a health behavior.

In summary, the purpose of this study was to examine whether perceived susceptibility and perceived severity positively predicted the level of young adults’ adherence to KDCA guidelines, as they have been proven to predict other health behaviors. Furthermore, this study examined sense of control as a moderator between the two constructs of HBM and adherence to COVID-19 prevention guidelines. More specifically, the following hypotheses were examined.

Hypothesis 1: Participants with a higher level of perceived susceptibility to COVID-19 are more likely to adhere to COVID-19 prevention guidelines.

Hypothesis 1-1: The effect of perceived susceptibility to COVID-19 on adherence to COVID-19 prevention guidelines is significantly moderated by sense of control.

Hypothesis 2: Participants with a higher level of perceived severity of COVID-19 are more likely to adhere to COVID-19 prevention guidelines.

Hypothesis 2-1: The effect of perceived severity of COVID-19 on adherence to COVID-19 prevention guidelines is significantly moderated by sense of control.

## Methods

### Participants

An online self-report survey targeting South Koreans between the ages of 19 and 29 (*M* = 24.505, *SD* = 2.669) was conducted between June 8th and 10th, 2021. According to Korea Disease Control and Prevention Agency (2021), on June 10th, the number of newly confirmed cases of COVID-19 was 611, and the number of cumulated confirmed cases was 144,152, which was approximately 2% of the entire population of South Korea. During this study period, COVID-19 prevention guidelines were applied at the city level according to the city’s number of new COVID-19 cases. The South Korean government implemented Level 2 social distancing rules, which was the strongest level at that time, in the Seoul capital area, and Level 1.5 social distancing rules in most other areas with a few exceptions such as the city of Daegu which also implemented Level 2 social distancing rules. Level 1.5 and Level 2 social distancing rules were similar. For example, under both rules, gatherings consisting of more than five people were not allowed. In public places such as theaters, stadiums, and libraries, people were asked to leave every other seat empty to practice social distancing. However, Level 1.5 rules did not require restaurants to restrict their operating hours and allowed them to accommodate more people in certain places such as sports stadiums and concert halls.

The minimum sample size for the moderate effect size (*f*^2^ = 0.15) was determined by G*power analysis. With α = 0.05, power = 0.80, the minimum sample size required was 146. Participants were recruited by convenience sampling with DataSpring Korea, a panel provider. DataSpring has a total of 416,962 South Korean panels, and among them, only the participants who responded that their nationality was South Korean and who were 19–29 years old were able to participate in the present study. Out of the 219 who initially participated 19 people were dropped (11 people did not meet the age requirement and eight people did not finish the survey) resulting in 200 participants’ responses being included in the final analysis. This study was approved by the Institutional Review Board of the researchers’ affiliated institution. Participants’ age, gender, marital status, education level, perceived economic status, city or province of residence, and perceived health status were collected as demographic variables (see Table 1). Participants were also asked about their COVID-19 self-quarantine and infection experiences and their family’s or friends’ infection experiences.

**TABLE 1:** Demographic information of participants.

Demographic	*N*	%
**Sex**		
Female	100	50
Male	100	50
**Educational background**		
Middle school graduate and below	2	1
High school graduate	100	50
University graduate	93	46.5
Graduate school graduate and above	5	2.5
**Marital status**		
Never married	191	95.5
Married	8	4
None of the above	1	0.5
**Perceived economic status**		
Very high	3	1.5
High	21	10.5
Middle	78	39
Low	71	35.5
Very low	27	13.5
**Perceived health status**		
Very good	28	14
Good	52	26
Average	90	45
Poor	29	14.5
Very poor	1	0.5
**Location**		
Area implementing Level 2 social distancing rules	137	68.5
Area implementing Level 1 social distancing rules	63	21.5
**COVID-19 self-quarantine experience** (due to history of oversea travel, close contacts with individual diagnosed with COVID-19)		
Yes	25	12.5
No	175	87.5
**COVID-19 diagnosis history (self)**		
Yes	0	0
No	200	100
**COVID-19 diagnosis history (family or friends)**		
Yes	22	11
No	178	89

### Measures

#### Perceived susceptibility and perceived severity of COVID-19

Wong et al. (2020) developed a questionnaire that measures COVID-19 infection and vaccination status that was derived from the five constructs of the HBM. Respondents responded with either “agree” or “disagree” for each item. In this study, only three items related to perceived susceptibility to and three items related to perceived severity of COVID-19 infection from the aforementioned scale were used. Participants responded on a five-point Likert scale where one corresponds to “strongly disagree” and five corresponds to “strongly agree” to allow participants to provide more information about their adherence behavior and adherence intentions. An example question of perceived susceptibility is “Chance of getting COVID-19 in the next few months is great” and of perceived severity is “Complications (that might result from COVID-19) are serious.” The Cronbach’s α for each construct was 0.73 and 0.83, respectively. All of the items were translated from English into Korean by a bilingual graduate psychology student and back-translated by another bilingual graduate psychology student.

#### Shapiro control inventory

Shapiro (1994) developed the Shapiro Control Inventory (SCI) which contains items measuring three areas: general domains of control, specific domains of control, and modes of control. The Korean version of this inventory (K-SCI) used in this study was validated by Sung and Park (2008). In this study, sense of control was measured using 14 items from the general domains of control, an area of which 10 were positive and four were negative. An example question of these items is “I am able to choose and make decisions about the important things in my life.” Participants responded to each item on a seven-point Likert scale. The negative control question was reverse-scored so that higher scores indicated a greater level of sense of control. The Cronbach’s α for this scale in this study was 0.78.

#### Adherence to Korean disease control and prevention agency guidelines

A questionnaire was developed for this study to determine the degree to which participants adhered to KDCA COVID-19 prevention guidelines. The KDCA provides two types of guidelines, i.e., general prevention guidelines and situation-specific prevention guidelines. Five items asked participants to rate the degree to which they had adhered to the general prevention over the preceding week, on a scale of 1–5 where one corresponds to “Never” and five corresponds to “Always.” With regards to situation-specific prevention guidelines, participants were only asked about their adherence to guidelines for visiting restaurants in the preceding week. However, at the time the survey was conducted, the KDCA required restaurants, cafes, and bars to close at 9 p.m. or 10 p.m., so it was unclear whether participants did not go to restaurants because they were willing to adhere to the guidelines, or simply because they were unable to go due to time restrictions. Thus, participants were asked about how willing they would be to adhere to prevention measures when visiting restaurants under Level 1 social distancing rules, which would not require restaurants to close early. There were 11 items that participants could respond to on the same five-point Likert scale as used for the general prevention guidelines items. Some of the items included in this questionnaire were “Washed hands thoroughly with soap and running water,” “Avoided visiting crowded places,” “[At a restaurant] wore the mask except for when eating food.” The entire questionnaire consisted of 16 items for which higher scores indicated better adherence. The Cronbach’α for all the 16 items was 0.90.

#### The center for epidemiologic studies depression scale

The Center for Epidemiologic Studies Depression Scale (CES-D) was developed by Radloff (1977) as a screening tool for depression by measuring symptoms and events experienced over the preceding week. It consists of 20 items, each of which can be responded to on a four-point Likert scale where zero corresponds to “Rarely or none of the time” and three corresponds to “All the time.” The Korean version of this scale (K-CES-D), which was validated by Chon et al. (2001), was used in this study. The Cronbach’s α for the questionnaire in this study was 0.86.

#### The generalized anxiety disorder questionnaire

The Generalized Anxiety Disorder questionnaire (GAD-7), developed by Spitzer et al. (2006), consists of seven items and rates the degree of perceived anxiety that the respondent felt during the preceding 2 weeks. Participants responded on a four-point Likert scale with zero corresponding to “Not at all” and three corresponding to “Nearly every day.” Seo and Park (2015) translated the Korean version of the questionnaire back into English and a native English speaker confirmed that the two versions were identical to each other. They also validated the Korean version of this questionnaire in patients with migraine. The Cronbach’s α for this questionnaire in this study was 0.91.

### Data analyses

Descriptive statistics for all the variables and correlations among the continuous variables were analyzed using IBM SPSS Statistics version 26. Pearson analysis was conducted to analyze the correlations. To determine whether sense of control moderated the relationship between the HBM constructs and adherence to COVID-19 prevention guidelines, hierarchical multiple regression analysis was conducted using a 5,000-replication bootstrap sample with a 95% bias-corrected CI (PROCESS 4.0, Model 1) (Hayes, 2021).

## Results

The result of the correlation analysis is presented in Table 2. Perceived susceptibility (*p* = 0.027), perceived severity (*p* < 0.001), and sense of control (*p* < 0.001) were positively correlated with adherence to prevention guidelines. Thus, Hypothesis 1 and Hypothesis 2 were supported. Depression was correlated with adherence to prevention guidelines, and thus depression and perceived health status were analyzed as covariates in the multiple regression analysis. Perceived health status was analyzed as covariates in the following multiple regression since there was a significant effect of perceived health status on adherence for the five levels of perceived health status, *F*(4, 195) = 2.584, *p* = 0.038.

**TABLE 2:** Correlations between variables.

Variables	*M*	*SD*	1	2	3	4	5	6
(1) Depression	22.485	12.495	—					
(2) Anxiety	13.16	5.299	0.821**	—				
(3) Perceived susceptibility	9.080	2.541	0.104	0.116	—			
(4) Perceived severity	11.415	2.746	–0.015	0.023	0.591**	—		
(5) Sense of control	60.855	11.934	−0.537**	−0.373**	0.051	0.182**	—	
(6) Adherence	62.470	10.315	−0.200**	–0.135	0.156*	0.303**	0.377**	—

*p < 0.05; **p < 0.01.

### Multiple regression analyses

Multiple regression analysis was conducted to determine whether sense of control moderated the relationship between perceived susceptibility and adherence to prevention guidelines (Table 3). The model containing perceived susceptibility, sense of control, and adherence to prevention guidelines was significant, *R*^2^ = 0.183, *F*(5, 194) = 8.705, *p* = 0.000. Both perceived susceptibility positively predicted adherence to prevention guidelines (β = 0.571, *SE* = 0.267, *t* = 2.140, *p* = 0.034, 95% CI [0.045, 1.098]) as did sense of control (β = 0.289, *SE* = 0.069, *t* = 4.205, *p* = 0.000, 95% CI [0.153, 0.424]) Depression (β = 0.000, *SE* = 0.067, *t* = 0.005, *p* = 0.996, 95% CI [−0.131, 0.132]) and perceived health status (β = −0.572, *SE* = 0.821, *t* = −0.697, *p* = 0.486, 95% CI [−2.191, 1.046]) did not predict adherence to prevention guidelines as covariates. The result also showed that sense of control significantly moderated the relationship between perceived susceptibility and adherence (β = −0.043, *SE* = 0.020, *t* = −2.176, *p* = 0.031, 95% CI [−0.083, −0.004]). The addition of the interaction between perceived susceptibility and sense of control was a significant change to the model, *R*^2^ = 0.020, *F*(1, 194) = 4.735, *p* = 0.031. Simple slope analysis (see Table 4) showed that perceived susceptibility positively predicted adherence to prevention guidelines at a sense of control 1 SD below the mean (β = 1.090, *SE* = 0.359, *t* = 3.032, *p* = 0.003, 95% CI [0.381, 1.798]), and at the mean level (β = 0.571, *SE* = 0.267, *t* = 2.140, *p* = 0.034, 95% CI [0.045, 1.098]). However, at a sense of control 1 SD above the mean, perceived susceptibility did not predict adherence to the prevention guidelines (β = 0.053, *SE* = 0.356, *t* = 0.150, *p* = 0.881, 95% CI [−0.649, 0.756]). This result demonstrates that perceived susceptibility to COVID-19 was positively correlated with adherence to prevention guidelines and that this relationship was stronger for those who had low and mean levels of sense of control. Thus, Hypothesis 1-1 was supported.

**TABLE 3:** Moderation effect of sense of control on the relationship between perceived susceptibility and adherence.

	*B*	*SE*	*t*	*p*	LLCI	ULCI
Perceived susceptibility	0.571	0.267	2.140	0.034	0.045	1.098
Sense of control	0.289	0.069	4.205	0.000	0.153	0.424
Interaction	–0.043	0.020	−2.176	0.031	–0.083	–0.004

**TABLE 4:** Conditional effects of perceived susceptibility at values of sense of control.

Sense of control	*B*	*SE*	*t*	*p*	LLCI	ULCI
−11.934	1.090	0.359	3.032	0.003	0.381	1.798
0.000	0.571	0.267	2.140	0.034	0.045	1.098
11.934	0.053	0.356	0.150	0.881	–0.649	0.756

**FIGURE 1:**
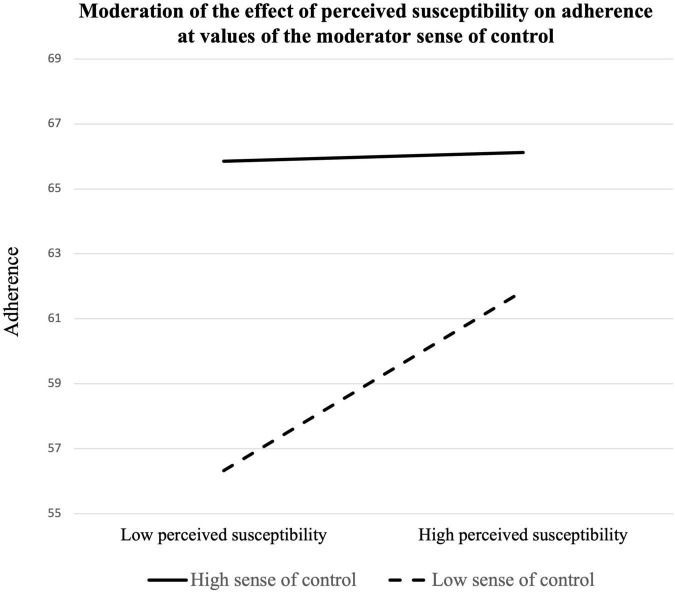
Moderation of the effect of perceived susceptibility on adherence at values of the moderator sense of control.

**FIGURE 2:**
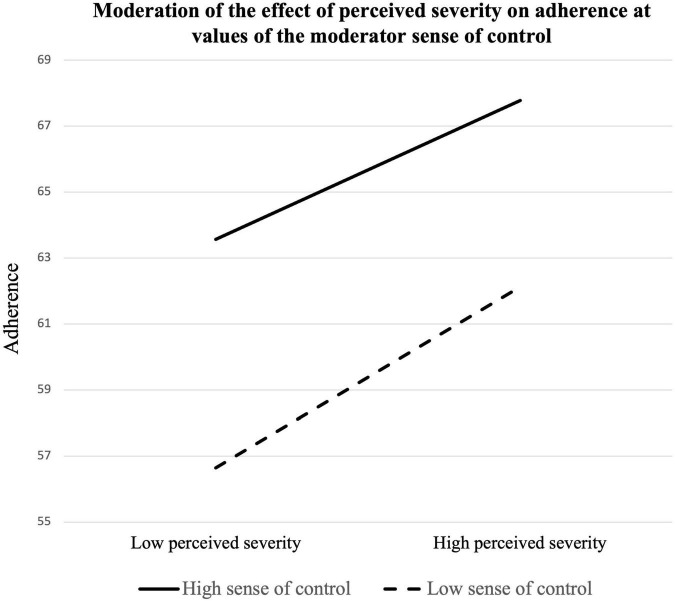
Moderation of the effect of perceived severity on adherence at values of the moderator sense of control.

Another multiple regression analysis was conducted to determine whether sense of control moderated the relationship between perceived severity and adherence to prevention guidelines (see Table 5). The model containing perceived severity, sense of control, and adherence behavior was significant, *R*^2^ = 0.203, *F*(5, 194) = 9.879, *p* = 0.000. Both perceived severity (β = 0.883, *SE* = 0.259, *t* = 3.407, *p* = 0.001, 95% CI [0.372, 1.395]) and sense of control (β = 0.263, *SE* = 0.069, *t* = 3.839, *p* = 0.000, 95% CI [0.128, 0.399]) positively predicted adherence to prevention guidelines. However, sense of control did not significantly moderate the relationship between perceived severity and adherence to prevention guidelines (β = −0.010, *SE* = 0.020, *t* = −0.475, *p* = 0.635, 95% CI [−0.050, 0.031]). Thus, Hypothesis 2-1 was not supported. The result of two moderation analyses is presented in Figures 1, 2.

**TABLE 5:** Moderation effect of sense of control on the relationship between perceived severity and adherence.

	*B*	*SE*	*t*	*p*	LLCI	ULCI
Perceived severity	0.883	0.259	3.407	0.001	0.372	1.395
Sense of control	0.263	0.069	3.839	0.000	0.128	0.399
Interaction	–0.010	0.020	−0.475	0.635	–0.050	0.031

## Discussion

In this study, young adults’ adherence to COVID-19 prevention guidelines was defined as a health behavior. This study examined how various factors predicted such adherence based on the HBM. In addition, as far as the authors know, this study is among the first to explore the relationship between sense of control and the HBM constructs by examining whether sense of control moderated the relationship between two HBM constructs, and health behavior/intention measured by adherence to COVID-19 prevention guidelines. The HBM constructs, namely perceived susceptibility, and perceived severity, were positively correlated with adherence to prevention guidelines, replicating past research on the HBM constructs predicting health behaviors (Bish and Michie, 2010; Liao et al., 2010; Lin et al., 2014; Venema and Pfattheicher, 2021).

The present study extends prior research by showing that sense of control positively predicts adherence to COVID-19 prevention guidelines. Correlation analysis showed that sense of control was the strongest predictor of adherence to prevention guidelines. Results of multiple regression analysis also showed that the main effect of sense of control on adherence behavior was significant in both two models as prior research had shown that sense of control was associated with better health and engagement in health behavior (Will Crescioni et al., 2011; Infurna et al., 2013; Infurna and Gerstorf, 2014; Hong et al., 2021; Precht et al., 2021).

This study’s findings further contribute to the literature showing that sense of control was correlated with the HBM constructs and sense of control. First, sense of control moderated the relationship between perceived susceptibility and adherence to prevention guidelines, supporting Hypothesis 1-1. Slope analysis showed that at a higher level of sense of control, the effect of perceived susceptibility was not correlated with adherence to prevention guidelines. This result indicates that while some individuals may think they are relatively less susceptible to COVID-19, they may still adhere to prevention guidelines if they feel strongly that they are able to control their environment. However, both at the lower and the mean levels of sense of control, the effect of perceived susceptibility was significant, which suggests perceived susceptibility also matters when individuals do not believe they have enough control over their environment. In contrast, the interaction effect was not significant between perceived severity and sense of control on adherence. While the interaction effect was not significant, the main effects of both perceived severity and sense of control on adherence were significant.

One possible explanation for this result is that perceived severity might have predicted the adherence to prevention guidelines better than perceived susceptibility when the survey was conducted. According to one past study, the two concepts can be distinguished by their relationship to the prevalence of a certain disease. More specifically, the prevalence of diseases such as flu, asthma, and cancer, was positively related to perceived susceptibility but negatively to perceived severity (El-Toukhy, 2015). Thus, the prevalence of COVID-19 at the time of the survey may have affected participants’ perceived susceptibility and perceived severity differently. When the survey was conducted in June 2021, in South Korea, the alpha and delta variants of COVID-19 were the dominant variants (Kim et al., 2022), which are considered to be more severe than Omicron, the dominant variant as of 2022. On June, 10th, 2021, there were 611 new confirmed cases of COVID-19 infection. Vaccination had begun around this time in South Korea, so the number of new cases was decreasing slightly but expected to decline even more significantly within a month (Korea Disease Control and Prevention Agency, 2021). This decline in the number of new cases may have caused participants to believe that they were less likely to get infected with COVID-19, thereby reducing their perceived susceptibility. However, it may not have had as big of an impact on their perceived severity because the concept of perceived severity assumes that someone has already contracted the disease and thus is unrelated to the likelihood of infection. This study’s results show that perceived severity was more strongly correlated with adherence to prevention guidelines than perceived susceptibility. Thus, when the survey was conducted, perceived severity may have had a greater effect on adherence to prevention guidelines than perceived susceptibility. This dynamic may explain why only perceived susceptibility was not correlated with adherence to prevention guidelines for those who felt a high sense of control.

### Implications

This study’s results have an implication for policymakers. In particular, since this study targeted individuals who are in their 20’s, the finding of this study may contribute to public messaging for improving adherence, specifically tailored for young adults. As young adults are known to have fewer concerns about COVID-19 infection, hospitalization, and death (Bechard et al., 2021), mere warnings about the severity of the symptoms or the long-term effects of infection might not be enough. Instead, policymakers may want to bolster people’s sense of control asit is a strong predictor of health behaviors and a protective factor against the negative influence of low perceived susceptibility. There are many strategies that policymakers can utilize to give people a better sense of control. Firstly, they might provide self-help manuals consisting of suitable techniques. Active confronting and reassuring thoughts, for example, increase sense of control which in turn improves psychological wellbeing (Dijkstra and Homan, 2016). Mindfulness techniques have also been shown to heighten sense of control (Pagnini et al., 2016). Also, meta-analysis results suggest that people who engage in self-help training in mindfulness/acceptance skills reported higher levels of proficiency and lower levels of anxiety and depression compared to the control group (Cavanagh et al., 2014). Applying this study’s results to developing self-help manuals in collaboration with clinical psychologists may help to improve individuals’ sense of control during the COVID-19 pandemic. Second, governments can help individuals gain a higher sense of control by providing the public with more information on COVID-19. A previous study has already proved that the more people believe they know about COVID-19, the happier they feel, and this relationship was mediated by sense of control (Yang and Ma, 2020). Lastly, the government can also promote the benefits of sense of control by managing the pandemic situation effectively. A disaster causes significant uncertainty, leading to decreased perceived control of the public (Afifi et al., 2014). The COVID-19 pandemic is distinguished from other disasters in that its progress and scales of damage can hardly be predicted (Osofsky et al., 2020). Some studies pointed out that uncontrollable circumstances may make a higher sense of control less adaptive than it normally is (Thompson et al., 1988; Heidemeier and Göritz, 2013). Thus, a higher sense of control of individuals alone might not be enough for promoting better behavioral adaptation during the pandemic. Rather, changes and adaptation at the governmental and organizational level might mitigate the uncontrollability of the COVID-19 situation and strengthen the advantage of a higher sense of control. A recent longitudinal study revealed that increased trust in the government dealing with the pandemic was related to higher self-reported adherence (Wright et al., 2021). In addition, organizations can also encourage their employees to adhere better to COVID-19 prevention guidelines by creating an organizational COVID-19 safety climate (Bazzoli and Probst, 2022; Hubert et al., 2022). As these studies suggest, individuals’ perceptions on how governments and organizations deal with the situation greatly influence their behavioral adaptation. Koffman et al. (2020) provided recommendations on how health professionals should handle uncertainties in the COVID-19 situation. For example, robust clinical trials for up-to-date treatments and developing guidelines constantly can be effective strategies both for government and health professionals to take. Taking extensive measures like these would mitigate the uncontrollability and uncertainty of the COVID-19 situation, thereby improving the individual’s sense of control and behavioral adaptation.

### Limitations and suggestions for future research

Firstly, one of the limitations of this study is that the required level of social distancing was not identical for all participants. More specifically, 21.5% of participants resided in areas that were subject to Level 1.5 social distancing rules, the measures comprising which were less severe than those faced by the other participants. Participants’ adherence to these guidelines may have been affected by how restrictive they were. Although there was no statistically significant difference in adherence to prevention guidelines by the level of social distancing rules, future studies should confirm whether people adhere differently to different levels of social distancing rules.

Next, we only measured two HBM constructs in the current study. However, other constructs should also be examined to determine how the sense of control relates to the whole HBM. The incremental theory of health or the belief that a particular aspect of one’s health can be changed through one’s efforts promoted engagement in health-protective behaviors during the COVID-19 pandemic (Zhang and Kou, 2021). This is related to the HBM’s perceived benefits construct which is defined as one’s belief in the efficacy of their behavior in reducing the risk or impact of contracting a disease (Glanz et al., 2008). As the severity of COVID-19 symptoms and its mortality rate is higher among the elderly than the rest of the population (Pepe et al., 2021), younger individuals may perceive the benefits of adhering to prevention guidelines differently from the elderly. Thus, future studies should examine how sense of control is correlated with each HBM construct, how these relationships differ between age groups and use a larger sample size.

Another limitation was that this study was cross-sectional in nature. Perceived susceptibility to COVID-19 increases over time (Shiloh et al., 2021), so adherence to prevention guidelines may change over time as a result. Longitudinal studies are needed to determine how the relationship between HBM constructs and health behaviors changes over time to reflect the changing COVID-19 situation.

In addition, because it was a self-reported questionnaire, the responses might not have been entirely reliable. Since adhering to KDCA guidelines is considered socially appropriate behavior for the pandemic situation, there is a possibility that the responses to the survey were distorted by social desirability bias (Jensen, 2020). According to a recent study, participants tended to overestimate their adherence to COVID-19 prevention guidelines (Mojzisch et al., 2022). As we were aware of this issue when creating the questionnaire, we clearly explained issues pertaining to confidentiality and data protection. We also emphasized the importance of participants’ honest responses in delineating the factors affecting adherence to COVID-19 prevention guidelines. These are all stated as strategies for reducing social desirability bias in a literature review (Krumpal, 2013). However, one recent study provided strategies in the context of COVID-19 (Timmons et al., 2021). For example, asking the number of behaviors engaged in from a given list of behaviors decreased the reported adherence compared to asking whether they engaged in each specific behavior. Future studies should examine whether this strategy can be replicated and effective in decreasing social desirability bias.

Lastly, there are some issues regarding the sample of this study. First, the sample size of the study is relatively small. Due to frequent shifts in South Korea’s social distancing policies (Seo et al., 2022), we attempted to collect the responses as fast as possible so that all participants could provide response under the same or at least similar social distancing policies. Thus, we collected the data in a very short period, which partly led to a small sample size. However, we conducted an *a priori* power analysis through the G*power program (Faul et al., 2007), and the minimum sample size required for obtaining a moderate effect size was 146. Since our sample size is 200, it is bigger than the minimum required sample size. Moreover, past studies on adherence to COVID-19 prevention guidelines with similar or slightly bigger sample sizes have also reported power analysis results to explain their sample size’s rationale (Xu and Cheng, 2021; Gul et al., 2022). Unlike these studies that collected responses from participants aged from 18 or 20 to 74, this study only targeted individuals in their 20’s. Because the heterogeneity of the sample caused by age is reduced in this study, this can compensate for the loss of power caused due to small sample size. Notwithstanding these points, we suggest future studies with a bigger sample size. Schönbrodt and Perugini (2013) recommended a sample size which is larger than 250 in order to have bigger statistical power and more stable result based on Monte-Carlo simulations. Second, regarding the representativeness of the sample, there was no participant who had experienced COVID-19 prior to participating in this study. This means that the obtained answers might not fully reflect the perceived susceptibility and severity level of the population they were intended to represent. However, in June 2021, when this study was conducted, approximately 2% of the whole population of South Korea had contracted COVID-19. As of November 2022, approximately 50% of the population has experienced COVID-19 infection. Thus, future study is needed to examine the difference between those who have and those who have not experienced COVID-19 infection. Furthermore, since this study conducted a web-based survey, there might have been a selection bias. This selection bias is crucial because people with limited access to the internet might not have been able to participate in the study (Bethlehem, 2010). However, according to the South Korean Ministry of Science and ICT (2022), the internet penetration rate was 81.9% in 2021, not including smartphones. Thus, it is not plausible that an under-representing issue might have occurred in this study. However, future studies are encouraged to consider the internet/smartphone penetration rate of the targeting population.

## Conclusion

This study’s results show that perceived susceptibility to COVID-19 infection, perceived severity of COVID-19, and sense of control positively predict adherence to prevention guidelines. The novelty of our study is in demonstrating the relationship between HBM constructs and sense of control in single model. The results show that sense of control can act as a buffer against the negative effect of lower perceived susceptibility to infection on adherence to prevention guidelines. These results have implications for public messaging intended to promote health behaviors in young adults during the COVID-19 pandemic, and interventions for those who do not strictly adhere to COVID-19 prevention guidelines.

## Data availability statement

The raw data supporting the conclusions of this article will be made available by the authors, without undue reservation.

## Ethics statement

The studies involving human participants were reviewed and approved by the Yonsei University Institutional Review Board. The ethics committee waived the requirement of written informed consent for participation.

## Author contributions

GL conceptualized and designed the research, collected, analyzed, and interpreted the data, and wrote the draft of the manuscript. SHP served as the principal investigator of the research grant and supervised the research process. Both authors provided critical feedback, participated in the revision of the manuscript, approved the final submission, and had full access to all data in the study and take full responsibility for the integrity of the data and the accuracy of the data analysis.
